# Assessment of MRI-Based Radiomics in Preoperative T Staging of Rectal Cancer: Comparison between Minimum and Maximum Delineation Methods

**DOI:** 10.1155/2021/5566885

**Published:** 2021-07-10

**Authors:** Haidi Lu, Yuan Yuan, Zhen Zhou, Xiaolu Ma, Fu Shen, Yuwei Xia, Jianping Lu

**Affiliations:** ^1^Department of Radiology, Changhai Hospital, No. 168 Changhai Road, Shanghai, China; ^2^Huiying Medical Technology Co., Ltd., B2, Dongsheng Science and Technology Park, HaiDian District, Beijing, China

## Abstract

The manual delineation of the lesion is mainly used as a conventional segmentation method, but it is subjective and has poor stability and repeatability. The purpose of this study is to validate the effect of a radiomics model based on MRI derived from two delineation methods in the preoperative T staging of patients with rectal cancer (RC). A total of 454 consecutive patients with pathologically confirmed RC who underwent preoperative MRI between January 2018 and December 2019 were retrospectively analyzed. RC patients were grouped according to whether the muscularis propria was penetrated. Two radiologists segmented lesions, respectively, by minimum delineation (Method 1) and maximum delineation (Method 2), after which radiomics features were extracted. Inter- and intraclass correlation coefficient (ICC) of all features was evaluated. After feature reduction, the support vector machine (SVM) was trained to build a prediction model. The diagnostic performances of models were determined by receiver operating characteristic (ROC) curves. Then, the areas under the curve (AUCs) were compared by the DeLong test. Decision curve analysis (DCA) was performed to evaluate clinical benefit. Finally, 317 patients were assessed, including 152 cases in the training set and 165 cases in the validation set. Moreover, 1288/1409 (91.4%) features of Method 1 and 1273/1409 (90.3%) features of Method 2 had good robustness (*P* < 0.05). The AUCs of Model 1 and Model 2 were 0.808 and 0.903 in the validation set, respectively (*P* = 0.035). DCA showed that the maximum delineation yielded more net benefit. MRI-based radiomics models derived from two segmentation methods demonstrated good performance in the preoperative T staging of RC. The minimum delineation had better stability in feature selection, while the maximum delineation method was more clinically beneficial.

## 1. Introduction

Rectal cancer (RC) is one of the most frequently diagnosed malignancies worldwide [1]. Accurate preoperative assessment of T staging of rectal cancer is a critical step in clinical treatment strategy, where a total mesorectal excision (TME) is considered as an optimal treatment approach for early staged RC (T1–2 and N-), while the treatment strategy for a locally advanced stage of RC (T3–4 and/or N+) is neoadjuvant chemotherapy (CRT) before TME [2, 3].

Currently, magnetic resonance imaging (MRI) is the common first-line modality for accurate pretreatment assessment of patients with RC. Moreover, rectal high-resolution T2-weighted images (T2WIs) have a vital role in the preoperative T staging of RC [3–5]. However, when there is an invasion of muscular layers by vessels, exudative changes around the lesion, and desmoplastic reaction, it is often hard to distinguish them from tumor infiltration outside the intestinal wall, which often leads to common mistakes in the staging of T2 and early T3 [4, 5].

Radiomics, a novel noninvasive tool, has shown multiple gratifying advantages in the preoperative assessment, prediction of treatment outcome, and distant metastasis of RC [6–10], thereby providing important details of tissue features, including the preoperative T staging. Among the factors that affect radiomics analysis, segmentation is vital as the first step of the imaging process. Still, recent publications have demonstrated that manual delineation of lesions is mainly used as a conventional segmentation method, but it is subjective and has poor stability and repeatability [9–11]. Zhang et al. [12] showed that delineation discrepancy in volumes of interest (VOIs) might affect predicting the performance of nasopharyngeal carcinoma and breast cancer radiomics models.

Some studies have reported on manual delineation based on MR images in RC patients. Most methodologies advocate using the volume of the whole primary tumor, which is manually drawn along the border of the tumor on each axial slice to cover the lesion [7, 8, 13–18]. Yet, most studies have no precise definition of the outer edge of the tumor. The type of manual segmentation method that can yield higher clinical benefit in patients with RC has been less discussed and requires further quantitative assessment. Therefore, the aim of our study was to validate and compare different radiomics tumor delineation models in evaluating the repeatability of feature extraction and exploring the preoperative T staging of RC based on high-resolution T2WI.

## 2. Materials and Methods

### 2.1. Participants

454 consecutive patients with RC who underwent 3.0 T rectal MRI before surgical resection at Changhai hospital between January 2018 and December 2019 were retrospectively assessed. Inclusion criteria were (1) pathologically confirmed RC with baseline MRI data, (2) baseline MRI within 14 days before surgical resection, and (3) single focus. Exclusion criteria were (1) a history of previous malignant tumor or pelvic surgery (*n* = 7), (2) poor quality of the images (*n* = 14), (3) received any treatment before and/or after baseline MR examination (*n* = 85), and (4) distant metastases (*n* = 31).

Based on the National Comprehensive Cancer Network (NCCN) and American Joint Committee on Cancer (AJCC) staging system [19], the patients were grouped according to different pathological T stages: T1–2 as a group without the penetrated muscularis propria and T3–4 as the group with penetration.

**Figure 1:**
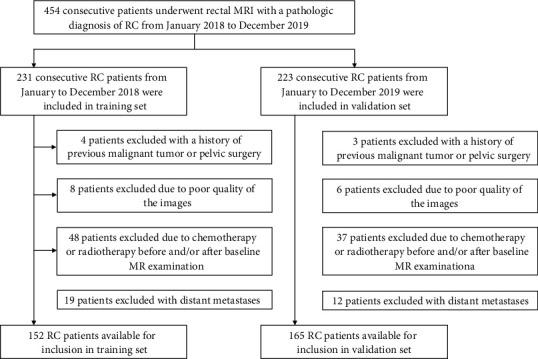
Diagram for the inclusion of patients into the study. RC: rectal cancer.

The training dataset and validation dataset were chronologically divided: 152 consecutive RC patients between January and December 2018 were included in the training set, while 165 consecutive RC patients between January 2019 and December 2019 were enrolled in the validation set (Figure 1).

The present study received approval from the local Institutional Review Board (Committee on Ethics of Biomedicine, Changhai Hospital). Informed consent was waived for this retrospective study.

### 2.2. Imaging Acquisition

Rectal MRI was scanned on two 3.0 T MR systems (Siemens Skyra 3.0T and GE Discovery 750w 3.0T) using a phased array coil. Before scanning, intestinal cleaning was performed by enema administration with 20 ml of glycerin. Oblique-axial high-resolution T2WI was perpendicular to the long axis of the rectum comprising the lesion. Routine sequences including sagittal T2WI, axial diffusion-weighted images (DWI, *b*-value: 0, 1000 s/mm^2^), axial T1-weighted images (T1WI), and gadolinium contrast-enhanced T1WI of the pelvis were obtained in the sagittal, coronal, and axial planes. Details on parameters applied for high-resolution T2WI, which were used for radiomics models, are shown in Supplemental Table 1.

### 2.3. Image Segmentation

All original high-resolution T2WI DICOM data were uploaded to the Huiying Medical Radcloud radiomics platform (http://radcloud.cn/). As the T2W images were required from two different MR systems in our study, image normalization was essential for all data to achieve homogeneity. Each image intensity was normalized to minimize the MRI signal variations using the following formula:

(1) $$\begin{array}{c} f\left(x\right)=\frac{s\left(x-\mu_{x}\right)}{\sigma_{x}}, \end{array}$$

where *f*(*x*) indicates the normalized intensity, *x* indicates the original intensity, *μ* refers to the mean value, *σ* indicates the variance, and *s* is an optional scaling, which is by default set to 1. While reserving the diagnostic intensity discrepancy, the signal discrepancy in MR parameters was decreased for subsequent radiomics analysis.

**Figure 2:**
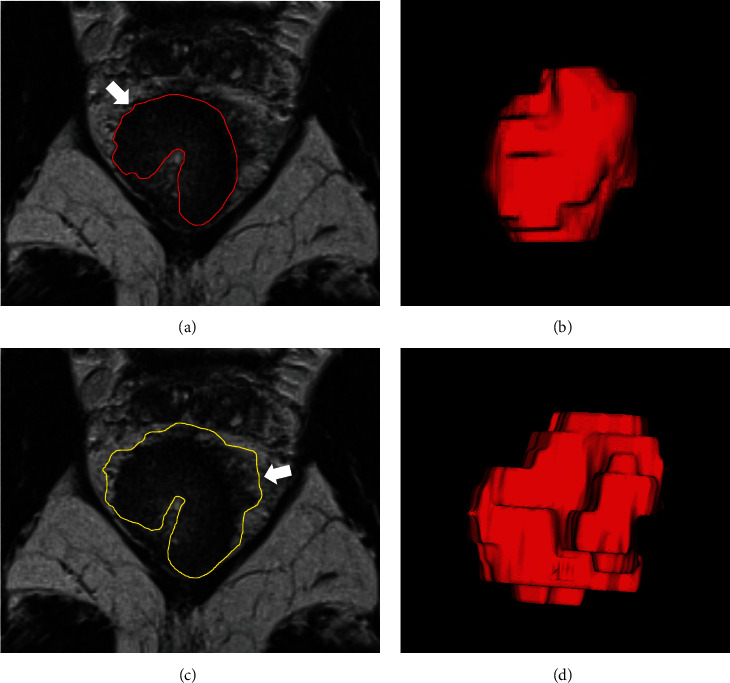
Representative images for lesion delineation. (a, b) Minimum delineation of ROI on oblique-axial T2-weighted MR images (arrow) and volume renderings of VOIs (Method 1). (c, d) Maximum delineation of ROI on oblique-axial T2-weighted MR images (arrow) and volume renderings of VOIs (Method 2).

The region of interest (ROI) of each lesion was manually delineated slice-by-slice on high-resolution T2W images. We used two kinds of manual segmentations for ROI: Method 1—minimum delineation and the smallest and clearest solid border that best fit the tumor region, excluding the blurry region of the margin; Method 2—maximum delineation, while the maximum margin of the lesion, including the entire region of perirectal tissues, was used to define the ROI (Figure 2). Then, the volume of interest (VOI) was reconstructed through the ROIs.

### 2.4. Feature Extraction and Reduction

Two radiologists with 8 (H.L.) and 5 years (Z.Z.) working experience in abdominal imaging independently reviewed all these images, who were blinded to the patient information. Next, all delineations were checked by one senior radiologist (Y.Y., who had 10 years of working experience in rectal MRI). Two radiologists (H.L. and Z.Z.) performed image processing of all cases on the platform, comprising Method 1 and Method 2, respectively. One radiologist (H.L.) repeated the segmentations of all cases one week later for final feature selection.

1409 radiomics features were extracted from each method of segmentation with the above platform. All features were grouped into four categories: (1) first-order features, which quantitatively delineated the distribution of voxel intensities of MR image by basic indexes; (2) shape-based features, including the shape and size of the VOI (e.g., the volume of segmentation); (3) texture features and quantification of the region heterogeneity differences; (4) higher order features, which included the transformation of first-order statistics and shape and texture characteristics, such as logarithm, exponential, gradient, square, square root, local binary patterns (LBP), and wavelet transformation [7, 8].

The inter- and intraclass correlation coefficient (ICC) was calculated to assess the reliability and reproducibility of all features. Features with both inter- and intraobserver ICCs exceeding 0.8 were applied for subsequent analysis, which suggested good robustness of features. To reduce the redundant features and select the optimal features, the variance threshold algorithm (variance threshold = 0.8) and Select-K-Best algorithm were adopted. The Select-K-Best algorithm used *P* < 0.05 to determine optimal features related to the T stage.

### 2.5. Machine Learning and Model Analysis

The radiomics analysis was performed in the Radcloud platform. Based on the selected features, the radiomics-based model was constructed with the support vector machine (SVM) in the training set, then verified in the validation set. For SVM, details of the parameters, kernel (linear), penalty coefficient (1), gamma (auto), class weight (balanced), decision function shape (one-to-many), and random state (NA), were used.

To assess the model's diagnostic performance, the receiver operator characteristic (ROC) curve was obtained by calculating areas under the curve (AUCs) in both datasets. The DeLong test was performed to evaluate differences between the ROC curves. The clinical benefits of radiomics models were estimated by decision curve analysis (DCA). Statistical significance was defined as *P* < 0.05.

## 3. Statistical Analysis

The Kolmogorov-Smirnov statistical test was used to test for the normality in all continuous variables. A paired Student's *t*-test or Wilcoxon test was used to compare variables between the two groups. Qualitative variables were assessed by the chi-square test or Fisher's exact test. SPSS software (version 20.0, Chicago, IL, USA) and R software (version 3.4.3) were used for statistical analysis. A *P* value of <0.05 was considered to be a statistically significant difference.

## 4. Results

### 4.1. Participant Characteristics

A total of 317 patients were finally enrolled. There was no significant difference between the training and validation sets. The patient characteristics and pathological outcomes are summarized in Table 1. According to the T stage by postoperative pathological examination, 183 patients (57.7%) were assigned to the penetration group.

### 4.2. Radiomics Features

All radiomics features extracted from Method 1 and Method 2 with ICCs ranged from 0.005 to 1.000. 1288/1409 features of Method 1 (91.4%) and 1273/1409 features of Method 2 (90.3%) had good robustness and were applied for subsequent analysis (both inter- and intraobserver ICCs ≥ 0.8). There was a significant statistical difference (*Z* = 18.574, *P* < 0.001) between the two methods.

The median (quartile range) volume of the two methods was 5.981 (2.490, 13.907) cm^3^ and 11.617 (5.594, 31.117) cm^3^, respectively. There was a significant difference in tumor size between Method 1 and Method 2 (*Z* = 3.29, *P* = 0.001).

Finally, 4 optimal features (Method 1) and 7 optimal features (Method 2) associated with T stage were selected to build the radiomics models (Model 1 and Model 2) (Table 2 and Supplemental Figure 1).

### 4.3. Performance of Radiomics Model

**Figure 3:**
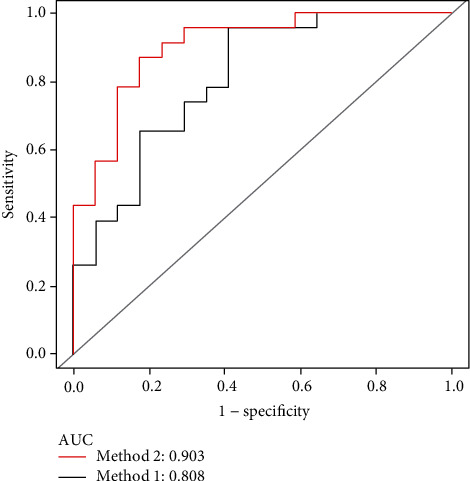
Receiver operator characteristic (ROC) curves in the validation set. AUC was 0.808 for the minimum delineation model (Method 1); AUC was 0.903 for the maximum delineation model (Method 2).

The ROC curves of the SVM classifier showed good performance with AUCs of 0.838 and 0.928 for Model 1 and Model 2 in the training set, respectively. For estimating differences in the two models in the validation set, Model 2 had an AUC of 0.903 (95% CI: 0.807-0.999), with a sensitivity of 87.0% and specificity of 82.3%, indicating a better performance compared with Model 1 that had an AUC of 0.808 (Figure 3). The DeLong test showed a significant difference (*P* = 0.035). Details contained in the models are shown in Table 3.

**Figure 4:**
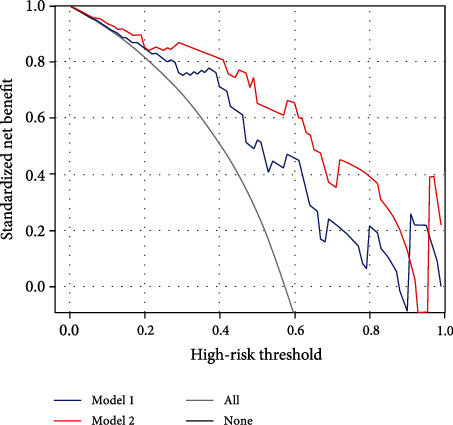
Decision curve analysis (DCA) of the two schemes of delineation. DCA showed that at the probability threshold of 0.0 to 0.9, the SVM model based on the maximum algorithm provided more net benefit than utilizing the minimum delineation scheme. Model 1: minimum delineation method; Model 2: maximum delineation method.

The decision curves demonstrated better performance of SVM models in predicting the T stage of RC than either the “all” or the “none” scheme at a threshold probability of 0.0-0.9 (Figure 4). The DCA showed that the Model 2 algorithm added more net benefit than that of Model 1.

## 5. Discussion

Our work showed that Method 2 had a better value in differentiating T1-2 from T3-4. Although the statistical difference was found between the two manual segmentations of MRI-based radiomics in ROC, Method 1 gained more stability and repeatability.

Due to the diverse treatments and prognoses, the distinction between T1-2 and T3-4 is quite important as it can prevent undertreatment or overtreatment. Among the widely used imaging methods, high-resolution MRI is the most commonly used imaging approach for this purpose. Even though rectal high-resolution T2WI is suggested for the conventional preoperative staging of RC, differentiation between T2 and early T3 tumors is still unsatisfactory [20, 21]. One common misunderstanding is caused by penetration to the muscular propria layers by small vessels and desmoplastic reaction, which may lead to a great challenge in staging by using traditional imaging methods [4, 5].

Previous academic studies have demonstrated that radiomics have good performance in evaluating many types of tumors and can be utilized as a profitable noninvasive modality for the local staging in RC [6–12]. The workflow involves acquisition and segmentation of images and extraction and reduction of features, and when the features are selected, a statistical model is established [10]. Among the factors that affect radiomics analysis, segmenting is essential as the imaging processing step. There are three segmentation methods: manual, semiautomatic, and automatic, each of which has its advantages and disadvantages. At present, manual delineation of the ROI is most commonly used as a conventional segmentation method; however, it is subjective and has poor stability and repeatability [22].

In the present study, two different manual segmentations were utilized to explore the influence of diverse delineation on the stability of feature selection and preoperative T staging's diagnostic efficiency. The inter- and intraclass correlation coefficients of features were computed. Our results showed that features based on minimum delineation had high robustness, which suggested good reliability and reproducibility.

Meanwhile, our results also showed that the diagnostic performance of radiomics models could be affected by delineation discrepancy. The above analysis indicated that the SVM model based on maximum delineation had a higher predictive performance than the minimum delineation model (*P* < 0.05) for T stage classification, thus suggesting good diagnostic efficiency. In their nasopharyngeal carcinoma and breast cancer studies, Zhang et al. [12] built a quantitative image postprocessing algorithm that demonstrated delineation differences in segmentation affecting radiomics-based diagnostic performance. Kocak et al. [23] analyzed the effect of radiomics segmentation with margin shrinkage in the evaluation of renal carcinomas. Nevertheless, manual segmentation tends to lead to the excessive delineation of the lesion border to ensure the entire lesion is recognized in most clinical practices [24]. Our clinical decision-making curves revealed that the clinical benefits of the maximum delineation algorithm were greater than the minimum approach in the evaluation of the T stage in RC patients, which is consistent with previous research [12, 25–29] and could be explained by the dilated margin of perirectal tissues containing complex information about identifying tumor heterogeneity.

This present study has several limitations. First, VOIs were manually delineated instead of being semiautomatically/automatically segmented, thus making it difficult to avoid subjective errors and making it unsuitable for large-scale data processing [30, 31]. Studies had indicated that semiautomated/automated segmentations can provide the reproducible and accurate estimates of the tumor [31–34]. However, similar to the previous studies, which used manual segmentation in RC patients, these studies described a semiautomated/automated delineated manner along the tumor's outer edge on each consecutive slice, with no precise definition of the border of the whole lesion. Second, this was a retrospective single-center cohort study without external validation. Therefore, a future multicenter study is required to verify our findings. Finally, we only discussed the effects of two manual segmentations of VOIs using T2WI. The effect of other routine sequences on diverse delineations, such as DWI and contrast-enhanced MRI, is still unclear and needs to be further investigated [35].

## 6. Conclusions

In this study, we developed two radiomics models based on different manual segmentations to assess the T stage in RC patients. The diverse delineation could cause certain differences in feature selection. Despite this discrepancy, both methods had good diagnostic performance in the preoperative T staging of RC. The minimum delineation had better stability in feature selection, while the maximum delineation was more beneficial in clinical decision-making.

## Figures and Tables

**Table 1:** Pathological characteristics of the patients.

Variables		Training set	Validation set	*P* value
		(*n* = 152)	(*n* = 165)	
Gender	Male	94 (61.8%)	109 (66.1%)	0.434
	Female	58 (38.2%)	56 (33.9%)	
Age (years)		58.9 ± 8.3	57.5 ± 8.8	0.147
BMI (kg/m^2^)		23.8 ± 3.2	23.5 ± 3.1	0.397
Tumor location	Upper	36 (23.7%)	32 (19.4%)	0.648
	Middle	92 (60.5%)	105 (63.6%)	
	Lower	24 (15.8%)	28 (17.0%)	
Histological type	Adenocarcinoma	131 (86.2%)	146 (88.5%)	0.325
	Mucinous adenocarcinoma	15 (9.9%)	17 (10.3%)	
	Signet ring cell carcinoma	6 (3.9%)	2 (1.2%)	
Differentiation	High	20 (13.2%)	17 (10.3%)	0.713
	Moderate	112 (73.7%)	127 (77.0%)	
	Poor	20 (13.2%)	21 (12.7%)	
T stage	T1	22 (14.5%)	17 (10.3%)	0.320
	T2	44 (28.9%)	51 (30.9%)	
	T3	74 (48.7%)	90 (54.5%)	
	T4	12 (7.9%)	7 (4.2%)	
N stage	N0	94 (61.8%)	99 (60.0%)	0.056
	N1	37 (24.3%)	28 (17.0%)	
	N2	21 (13.8%)	38 (23.0%)	
Tumor deposit	Negative	118 (77.6%)	137 (83.0%)	0.226
	Positive	34 (22.4%)	28 (17.0%)	
Lymphovascular invasion	Negative	91 (59.9%)	100 (60.6%)	0.893
	Positive	61 (40.1%)	65 (39.4%)	
Perineural invasion	Negative	106 (69.7%)	117 (70.9%)	0.819
	Positive	46 (30.3%)	48 (29.1%)	
Tumor budding	Negative	114 (75.0%)	126 (76.4%)	0.777
	Positive	38 (25.0%)	39 (23.6%)	
CEA^∗^	Negative	107 (70.4%)	115 (69.7%)	0.892
	Positive	45 (29.6%)	50 (30.3%)	
CA19-9^∗^	Negative	126 (82.9%)	126 (76.4%)	0.150
	Positive	26 (17.1%)	39 (23.6%)	

BMI: body mass index; CEA: carcinoembryonic antigen; CA19-9: carbohydrate antigen 19-9. ∗Preoperative blood samples.

**Table 2:** Selected radiomics features.

Model	No	Radiomics feature	Radiomics class	Filter
Method 1	1	Skewness	First order	Wavelet-HLL^∗^
	2	Maximum	First order	Wavelet-HLL^∗^
	3	High gray level zone emphasis	GLSZM	Wavelet-HLH^∗^
	4	Gray level nonuniformity	GLSZM	Wavelet-LHL^∗^
Method 2	1	Skewness	First order	Wavelet-HLL^∗^
	2	High gray level zone emphasis	GLSZM	Wavelet-LHL^∗^
	3	Skewness	First order	Wavelet-LHL^∗^
	4	High gray level run emphasis	GLRLM	Original
	5	High gray level run emphasis	GLRLM	Logarithm
	6	High gray level run emphasis	GLRLM	Square root
	7	High gray level run emphasis	GLRLM	Wavelet-LLL^∗^

GLSZM: gray level size zone matrix; GLRLM: gray level run length matrix. ^∗^The wavelet transform decomposes the tumor area image into low-frequency components (L) or high-frequency components (H) in the *x*, *y*, and *z* axes. Method 1: minimum delineation method; Method 2: maximum delineation method.

**Table 3:** ROC analysis of the prediction model for the training and validation sets.

	Training set		Validation set	
	Method 1	Method 2	Method 1	Method 2
AUC	0.838	0.928	0.808	0.903
95% CI	0.764-0.912	0.864-0.992	0.669-0.947	0.807-0.999
Sensitivity	0.871	0.903	0.956	0.870
Specificity	0.805	0.866	0.588	0.823
Accuracy	0.823	0.876	0.800	0.850
PLR	4.464	6.733	2.323	4.927
NLR	0.160	0.112	0.074	0.158
PPV	0.628	0.718	0.759	0.870
NPV	0.943	0.960	0.909	0.823
*P* ^∗^	0.036		0.035	

PLR: positive likelihood ratio; NLR: negative likelihood ratio; NPV: negative predictive value; PPV: positive predictive value. ^∗^Compared by DeLong test.

## Data Availability

The datasets used and/or analyzed during the current study are available from the corresponding author on reasonable request.

## Abbreviations

RC: Rectal cancer
MRI: Magnetic resonance imaging
AUCs: Areas under the curves
TME: Total mesorectal excision
ICC: Intraclass correlation coefficient
ROC: Receiver operator characteristic
DCA: Decision curve analysis
T2WI: T2-weighted imaging
ROI: Region of interest
VOI: Volume of interest
SVM: Support vector machine
TR: Repetition time
TE: Time of echo
DWI: Diffusion weighted imaging.

## Additional Points

*STARD Checklist*. The checklist according to STARD 2015 guidelines for reporting diagnostic accuracy studies.

## Supplementary Materials

Supplementary Materials Supplemental Table 1: details on parameters applied for high-resolution T2WI, which were used for radiomics models. Supplemental Figure 1: the diagram of feature extraction. A and B: we used the variance threshold method to select 441 features (A: minimum delineation) and 444 features (B: maximum delineation) from 1409 features, respectively. C and D: We used select *K*-best methods to further select radiomics features. Finally, 4 optimal features (C: minimum delineation) and 7 optimal features (D: maximum delineation) were selected.
