# Supplementary material for: Assessment of MRI-Based Radiomics in Preoperative T Staging of Rectal Cancer: Comparison between Minimum and Maximum Delineation Methods
Source: Biomed Res Int. 2021 Jul 10;2021:5566885. doi: 10.1155/2021/5566885 (PMC8289571; doi:10.1155/2021/5566885)
Supplement: Supplementary Materials — Supplemental Table 1: details on parameters applied for high-resolution T2WI, which were used for radiomics models. Supplemental Figure 1: the diagram of feature extraction. A and B: we used the variance threshold method to select 441 features (A: minimum delineation) and 444 features (B: maximum delineation) from 1409 features, respectively. C and D: We used select K-best methods to further select radiomics features. Finally, 4 optimal features (C: minimum delineation) and 7 optimal features (D: maximum delineation) were selected. [file 5566885.f1.zip › 5566885.f1.docx]

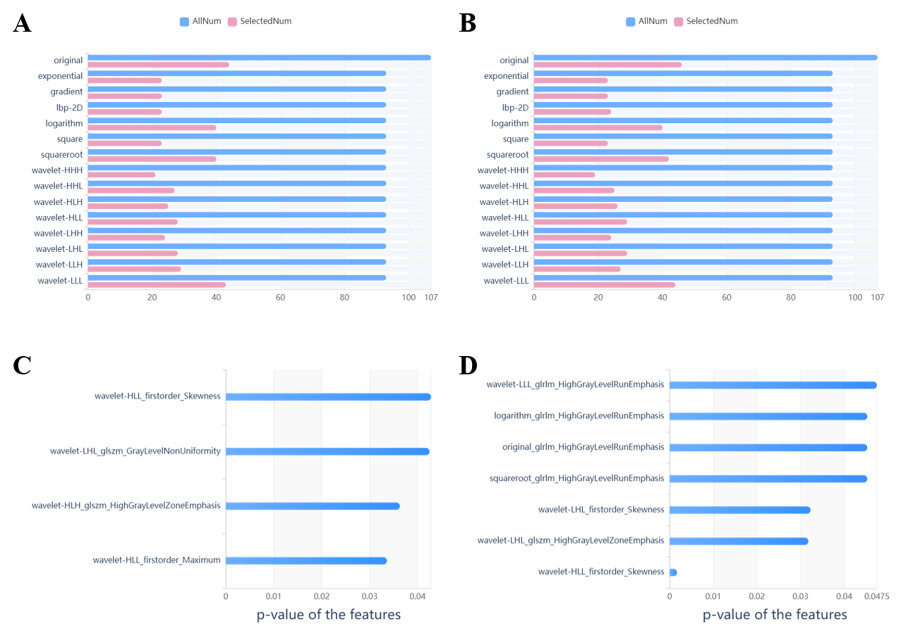


**Supplemental Figure 1.** The diagram of feature extraction. A and B: We used variance threshold method to select 441 features (A: minimum delineation) and 444 features (B: maximum delineation) from 1409 features, respectively. C and D: We used Select K best methods to further select radiomics features. Finally, 4 optimal features (C: minimum delineation) and 7 optimal features (D: maximum delineation) were selected.
