# Supplementary material for: Assessment of MRI-Based Radiomics in Preoperative T Staging of Rectal Cancer: Comparison between Minimum and Maximum Delineation Methods
Source: Biomed Res Int. 2021 Jul 10;2021:5566885. doi: 10.1155/2021/5566885 (PMC8289571; doi:10.1155/2021/5566885)
Supplement: Supplementary Materials — Supplemental Table 1: details on parameters applied for high-resolution T2WI, which were used for radiomics models. Supplemental Figure 1: the diagram of feature extraction. A and B: we used the variance threshold method to select 441 features (A: minimum delineation) and 444 features (B: maximum delineation) from 1409 features, respectively. C and D: We used select K-best methods to further select radiomics features. Finally, 4 optimal features (C: minimum delineation) and 7 optimal features (D: maximum delineation) were selected. [file 5566885.f1.zip › 5566885.f2.docx]

**Supplemental Table 1. High-resolution T2WI sequence acquisition parameters**

|  | Sequence name | TR/TE (m/s) | Echo train length | Matrix | FOV  (mm) | Slice thickness/gap (mm) | Bandwidth (Hz) /FA (°) | Acquisition times |
| --- | --- | --- | --- | --- | --- | --- | --- | --- |
| **SIEMENS**  Magnetom Skyra | TSE | 4000/108 | 16 | 320×320 | 180×180 | 3/0 | 108/160 | 4 min 10 sec |
| **GE**  Discovery 750w | FSE | 6538/116 | 32 | 352×352 | 200×200 | 3/0 | 62.5/110 | 3 min 16 sec |

TSE: Turbo spin echo; FSE: Fast spin echo; TR/TE: Repetition time/echo time; FOV: Field of view; FA: Flip angle.
